# The Relationship Between Opioid Dose and Opioid-Induced Constipation in Japanese Patients With Cancer Pain: A Post Hoc Analysis of a Prospective Observational Cohort Study

**DOI:** 10.7759/cureus.87697

**Published:** 2025-07-10

**Authors:** Soichi Fumita, Hisao Imai, Toshiyuki Harada, Yusaku Akashi, Yuichi Koretaka, Yasuhide Morioka, Yoshiyuki Kizawa, Akihiro Tokoro

**Affiliations:** 1 Department of Medical Oncology, Kindai University Nara Hospital, Ikoma, JPN; 2 Department of Respiratory Medicine, Comprehensive Cancer Center, Saitama Medical University International Medical Center, Hidaka, JPN; 3 Division of Respiratory Medicine, Gunma Prefectural Cancer Center, Ota, JPN; 4 Department of Respiratory Medicine, Center for Respiratory Diseases, Japan Community Healthcare Organization Hokkaido Hospital, Sapporo, JPN; 5 Department of Data Science, Shionogi & Co. Ltd., Osaka, JPN; 6 Department of Medical Affairs, Shionogi & Co. Ltd., Osaka, JPN; 7 Department of Palliative and Supportive Care, Institute of Medicine, University of Tsukuba, Tsukuba, JPN; 8 Department of Psychosomatic Internal Medicine and Supportive and Palliative Care Team, National Hospital Organization Kinki-Chuo Chest Medical Center, Sakai, JPN

**Keywords:** bowel function index, cancer, daily opioid dose, opioid, opioid-induced constipation, post hoc analysis, risk factors, rome iv

## Abstract

Introduction: This is a post hoc analysis of the multicenter, prospective, observational cohort study of Opioid-Induced Constipation in patients with cancer pain in Japan (OIC‑J) study (UMIN000025864), which investigated the incidence of opioid-induced constipation (OIC) in patients with cancer. The objective of the present study was to explore the relationship between opioid dose and the development of OIC.

Methods: Patients of either sex, aged ≥20 years, with cancer pain and an Eastern Cooperative Oncology Group performance status (ECOG PS) score of two or less, and who had no pre‑existing constipation and required initiation of strong opioid analgesics were included. Patients who started laxatives on the same day of opioid initiation were considered to have “prophylactic use of laxatives” and were excluded. The relationship between daily opioid dose (oral morphine milligram equivalent; MME) and OIC incidence was assessed, along with Bowel Function Index (BFI) scores.

Results: A total of 100 patients without prophylactic laxative use were included. Although none of the patients had constipation before opioid initiation, 66% developed OIC within two weeks of opioid initiation. Daily opioid dose was compared between the OIC group (n=66) and the non-OIC group (n=34) to determine whether opioid use differed based on the presence or absence of OIC. The mean±standard deviation daily opioid dose was higher in the OIC group (22.5±15.7 MME/day) than in the non-OIC group (17.8±13.0 MME/day), although the difference was not statistically significant (*p*=0.1380). When assessing the trend between opioid dose and OIC incidence, the proportion of patients developing OIC increased numerically with higher opioid doses from 54.2% (95% confidence interval [CI]: 32.8‑74.5) at ≤10 mg/day to 80.8% (95% CI: 44.4-97.5) at >40-80 mg/day. In the multivariate logistic regression analysis, the odds ratio for daily opioid dose (per 10 mg) was 1.339 (95% CI: 0.949-1.890), indicating a trend toward increased OIC incidence with higher opioid doses; however, this was not a statistically significant factor associated with OIC incidence (*p*=0.096). Furthermore, no correlation was observed between opioid dose and BFI score (*r*=0.258).

Conclusions: This post hoc analysis reported that OIC incidence is not significantly dependent on opioid dose. The high incidence of OIC even in patients with cancer receiving low-dose opioids highlights the need to manage OIC proactively, regardless of the opioid dose.

## Introduction

Opioids are frequently used for effective pain management in patients with cancer due to their potent analgesic effects; however, they are commonly associated with various gastrointestinal side effects, particularly opioid-induced constipation (OIC) [1-3].

OIC is characterized by the presence of hard stools or difficulty in passing stools, straining during defecation, and a sensation of incomplete evacuation or anorectal obstruction following the initiation of opioid treatment [4,5]. A real-world, multicenter, observational, large cohort study (n=1,000) reported that approximately 59% of patients with cancer experienced OIC [6]. Another multicenter, prospective, observational cohort study conducted in Japanese patients with cancer pain initiating strong opioids reported an OIC incidence of 61% [4]. Overall, the incidence of OIC in patients with cancer receiving opioids ranges between 40% and 60% [7]. The prevalence of OIC tends to increase with the duration of opioid treatment [3], reaching up to 90% in patients with advanced cancer [7]. Furthermore, patients with cancer are more likely to report constipation as a source of distress [3], highlighting its substantial impact on patients’ quality of life [6,8,9].

The first-line treatment for OIC includes exercise, use of stimulant laxatives, increased dietary fiber intake, and increased hydration [5,10]. However, these approaches often offer inadequate or inconsistent relief [10]. Although several reports have suggested no correlation between opioid dose and the onset of OIC [6,11,12], some patients reduce or discontinue their opioid use in an attempt to manage constipation and avoid OIC, often at the cost of adequate pain control [1]. However, studies specifically assessing the relationship between opioid dose and the occurrence of OIC remain limited.

In the observational Opioid-Induced Constipation in patients with cancer pain in Japan (OIC‑J) study, Tokoro et al. investigated the incidence of OIC in patients with cancer [4]. To address the existing knowledge gap, we conducted a post hoc subgroup analysis of the OIC‑J study, focusing on the relationship between opioid dose and the development of OIC. We also hypothesized that higher opioid doses may be linked to an increased incidence of OIC in patients with cancer pain.

## Materials and methods

This was a retrospective, post hoc analysis conducted using data from the OIC-J study, which was a multicenter, prospective, observational cohort study, the primary and secondary analysis results of which have been published previously [4]. The OIC-J study was conducted between January 5, 2017, and January 31, 2018, at 28 medical institutions across Japan and evaluated the incidence of OIC using the Rome IV diagnostic criteria in patients with cancer pain initiating strong opioid therapy. It was approved by the Institutional Review Board of the National Hospital Organization Kinki‐Chuo Chest Medical Center (approval number: 28-18), and registered with the University Hospital Medical Information Network Clinical Trials Registry (UMIN-CTR) (registration number: UMIN000025864).

Participants

The patient selection criteria have been described previously [4]. Briefly, patients of either sex, aged ≥20 years, with cancer pain and an Eastern Cooperative Oncology Group performance status (ECOG PS) score of ≤2, with an expected stable cancer condition throughout the study, and who had no pre-existing constipation and required initiation of opioid analgesics were included. Specifically, patients with three or more bowel movements in the seven days before enrollment were included in the analysis. Patients were excluded if they had conditions affecting gastrointestinal tract structure or function; had undergone related surgery, interventions, or radiotherapy within 28 days before enrollment or during the study period; or had undergone disimpaction within seven days before or during the study [4].

Additionally, for this post hoc analysis, patients who started laxatives on the same day of opioid initiation were considered as having “prophylactic use of laxatives” and were excluded.

Data collection

Following the initiation of opioids, patients used a paper diary to record their bowel habits. Every day, the patients recorded the date and time of each bowel movement, constipation symptoms as defined by Rome IV diagnostic criteria [13], as well as type, time, and dose of both rescue (if any) and scheduled opioid administrations. The paper-based diary entries were verified by the investigator or their designee and subsequently entered into the electronic data capture system [4].

Outcomes

The presence and degree of correlation between opioid dose and the incidence of OIC within two weeks of initiating strong opioid therapy were analyzed, using the Rome IV diagnostic criteria for OIC [4]. OIC is defined as new or worsening symptoms of constipation when initiating, changing, or increasing opioid therapy that must include two or more of the following occurring in more than 25% of defecations: straining, lumpy or hard stools (Bristol Stool Form Scale 1-2 [14]), sensation of incomplete evacuation, sensation of anorectal obstruction/blockage, manual maneuvers, fewer than three spontaneous bowel movements per week. In addition, the relationship between opioid dose and the Bowel Function Index (BFI) score [15], a patient-reported measure of bowel function (in patients undergoing opioid therapy), ranging from 0 to 100, was assessed. The BFI score was defined as the highest score recorded at one or two weeks after opioid initiation; a score above 28.8 was considered indicative of constipation.

The total amount of scheduled and rescue opioids administered during the observation period was converted to the oral morphine equivalent dose and then transformed into the daily dose. This value was used as the opioid dose (oral morphine milligram equivalent; MME/day).

Statistical analyses

All statistical analyses were performed using SAS version 9.4 (SAS Institute Inc., Cary, North Carolina, United States). Continuous variables were summarized as mean±standard deviation (SD), median (first quartile [Q1]-third quartile [Q3]), minimum, and maximum values. Differences between the OIC and non-OIC groups were compared using the t-test. Categorical variables were summarized as frequencies and proportions with 95% confidence intervals (CIs). Risk factors such as sex, age, performance status, anticancer medication, and comorbidities were selected based on the results from the OIC-J study [4]. Additionally, opioid dose, not assessed in the OIC-J study but assessed in the present study, was also used as a risk factor. Logistic regression analysis was performed to identify the association between these factors and OIC incidence. The correlation between opioid dose and BFI scores was determined using Pearson’s correlation coefficient. A two-sided *p*-value of <0.05 was considered statistically significant.

## Results

Patient disposition

There were a total of 208 patients who met the initial inclusion criteria for the OIC-J study [4]. Of these, 100 patients who had not taken any laxatives for prophylactic use and had an observation period of eight or more days were included in this post hoc analysis. Finally, 66 patients were included in the OIC group and 34 patients in the non-OIC group of the present study.

Opioid dose

**Figure 1:**
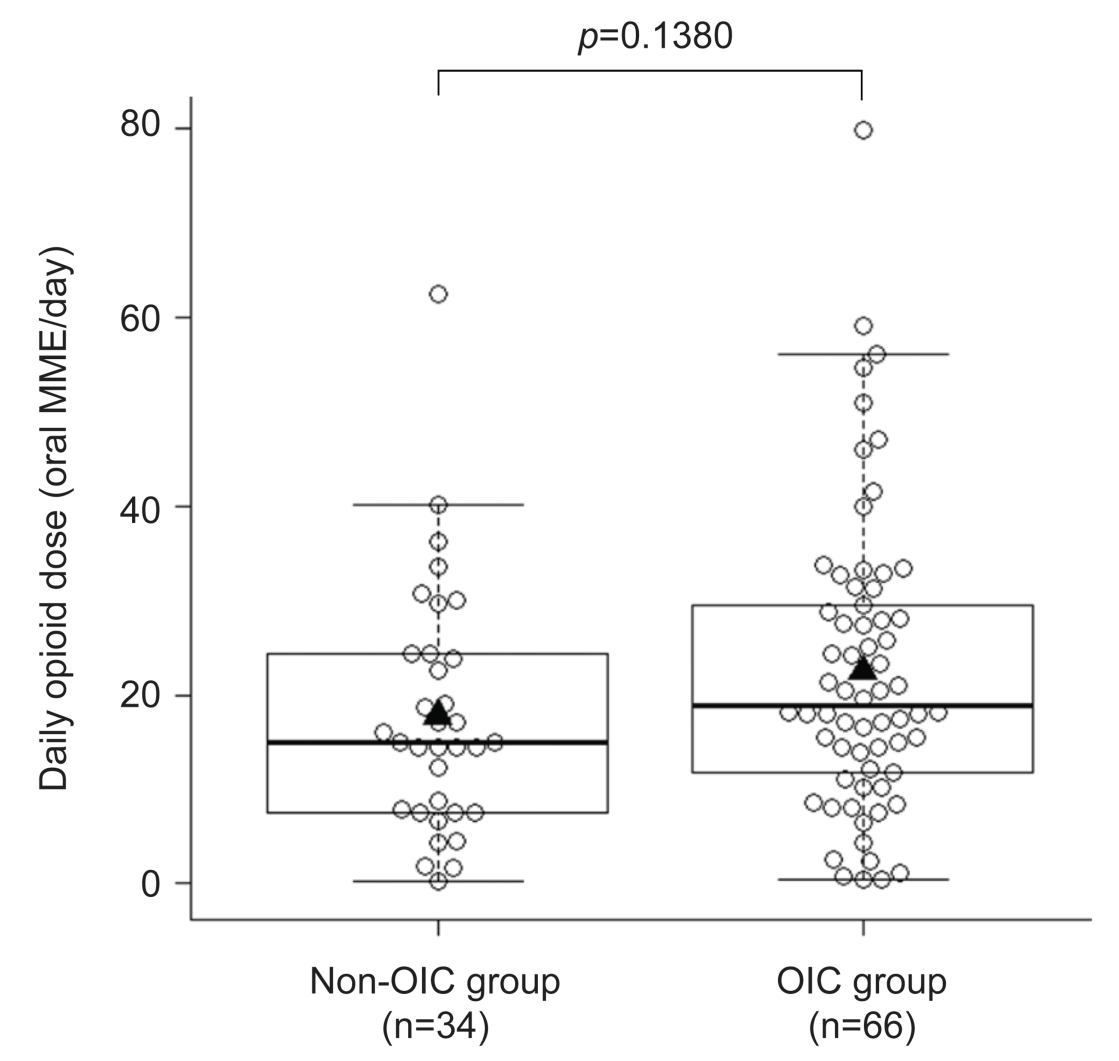
Opioid dose in patients with cancer pain who developed OIC and those who did not The *p*-value was calculated using the t-test. ▲ indicates mean values. The box plot shows the median (Q1-Q3). MME, morphine milligram equivalent; OIC, opioid‑induced constipation; Q1, first quartile; Q3, third quartile

The mean±SD opioid doses in the OIC and non-OIC groups were 22.5±15.7 MME/day and 17.8±13.0 MME/day, respectively. The median (Q1-Q3) opioid doses in the OIC and non‑OIC groups were 18.9 (11.8-29.5) MME/day and 15.0 (7.5-24.4) MME/day, respectively. Although the OIC group tended to have a slightly higher distribution of opioid doses, the difference was not statistically significant (*p*=0.1380) (Figure 1 and Table 1).

**Table 1:** Opioid dose (oral MME/day) in patients with cancer pain who developed OIC and those who did not Max, maximum value; Min, minimum value; MME, morphine milligram equivalent; OIC, opioid‑induced constipation; Q1, first quartile; Q3, third quartile; SD, standard deviation.

	Non-OIC group (n=34)	OIC group (n=66)
Mean (SD)	17.8 (13.0)	22.5 (15.7)
Median (Q1-Q3)	15.0 (7.5-24.4)	18.9 (11.8-29.5)
Min-Max	0.3-62.5	0.5-79.8

Opioid dose and proportion of OIC incidence

**Figure 2:**
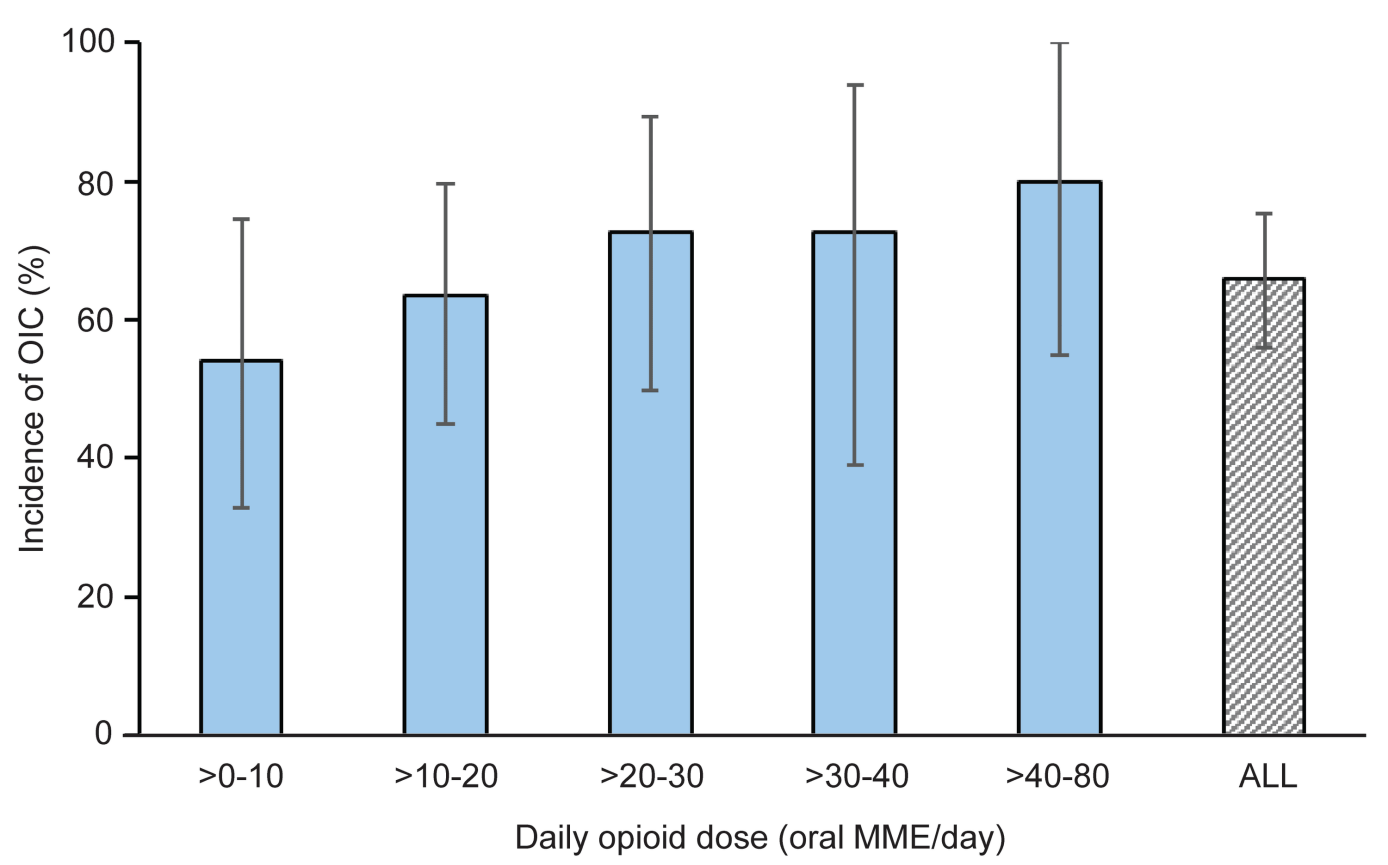
Opioid dose and OIC incidence in patients with cancer pain (N=100) MME, morphine milligram equivalent; OIC, opioid-induced constipation.

The incidence of OIC in all patients was 66.0% (95% CI: 55.9-75.2). The incidence rate of OIC was evaluated for every 10 mg/day increment in opioid dose. The incidence rate of OIC was 54.2% (95% CI: 32.8-74.5; n=24) for patients taking ≤10 mg/day of opioid. As the opioid dose increased, the proportion of patients with OIC also increased numerically. Among patients receiving an opioid dose of >40-80 mg/day, 80.8% (95% CI: 44.4-97.5) developed OIC; however, only 10 patients were taking opioids at a dose of >40-80 mg/day, making this the smallest group (Figure 2 and Table 2).

**Table 2:** Opioid dose and OIC incidence in patients with cancer pain CI, confidence interval; MME, morphine milligram equivalent; OIC, opioid-induced constipation.

Daily opioid dose (oral MME/day)	>0-10	>10-20	>20-30	>30-40	>40-80	Total
Number of patients	24	33	22	11	10	100
OIC, % (n)	54.2% (13)	63.6% (21)	72.7% (16)	72.7% (8)	80.8% (8)	66.0% (66)
95% CI	32.8-74.5	45.1-79.6	49.8-89.3	39.0-94.0	44.4-97.5	55.9-75.2

Adjusted impact of opioid dose on the incidence of OIC

The adjusted odds ratio (OR) for daily opioid dose (per 10 mg), adjusted for sex, age, performance status, anticancer medication, and comorbidities, was 1.339 (95% CI: 0.949-1.890). The daily opioid dose was not a statistically significant factor (*p*=0.096). None of the other factors assessed were associated with the incidence of OIC (Table 3).

**Table 3:** Risk factors for the incidence of OIC *Entered into the logistic regression analysis as continuous variables and converted to units of 10 mg/day. Logistic regression analysis was performed using logistic procedure for SAS 9.4 (SAS Institute Inc., Cary, NC) to identify the association between relevant risk factors (sex, age, performance status, anticancer medication, comorbidities, and daily opioid dose) and OIC incidence. CI, confidence interval; OIC, opioid-induced constipation.

Characteristics		OIC group (n=66), n (%)	Non-OIC group (n=34), n (%)	Odds ratio (95% CI)	*p*-value
Sex	Male	42 (63.6)	22 (64.7)	Ref	0.741
	Female	24 (36.4)	12 (35.3)	0.842 (0.303-2.336)	
Age (years)	<75	52 (78.8)	22 (64.7)	Ref	0.125
	≥75	14 (21.2)	12 (35.3)	0.453 (0.165-1.246)	
Performance status	<2 (0-1)	50 (75.8)	28 (82.4)	Ref	0.301
	≥2 (2)	16 (24.2)	6 (17.6)	1.875 (0.569-6.175)	
Anticancer medication	No	28 (42.4)	20 (58.8)	Ref	0.097
	Yes	38 (57.6)	14 (41.2)	2.187 (0.868-5.507)	
Comorbidities	No	26 (39.4)	8 (23.5)	Ref	0.131
	Yes	40 (60.6)	26 (76.5)	0.464 (0.171-1.257)	
Daily opioid dose (per 10 mg/day)		-	-	1.339 (0.949-1.890)*	0.096

Opioid dose and BFI score

**Figure 3:**
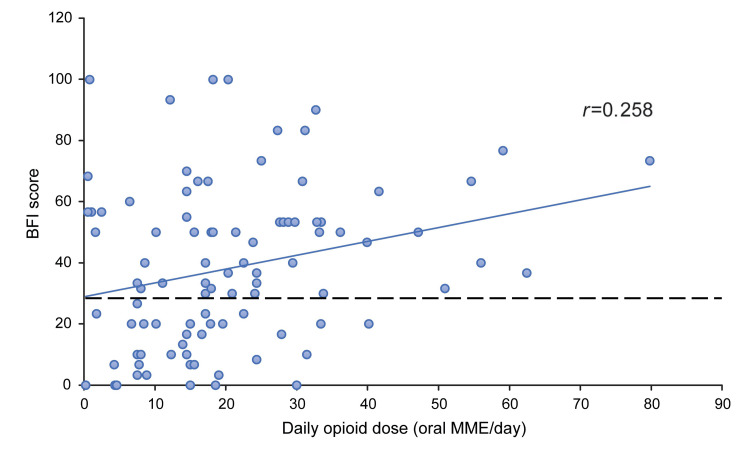
Opioid dose and BFI score in patients with cancer pain The BFI score [15], a patient-reported measure of bowel function ranging from 0 to 100, was defined as the highest score recorded at one or two weeks after opioid initiation. The score above 28.8 (dotted line) was considered indicative of constipation. The correlation between the daily opioid dose and BFI scores was determined using Pearson's correlation coefficient. BFI, Bowel Function Index; MME, morphine milligram equivalent

The correlation between opioid dose and BFI scores was plotted. There was no correlation (*r*=0.258) between opioid dose and BFI scores, as seen in the scatter plot (Figure 3).

## Discussion

The present study is the first study to provide a detailed analysis of the relationship between opioid dose and the development of OIC, diagnosed using the Rome IV diagnostic criteria, in Japanese patients with cancer pain. The study is a post hoc analysis of a subgroup from a previous observational study (the OIC-J study), which investigated the incidence of OIC in this population [4].

In this analysis, we compared opioid dose distributions between patients who developed OIC and those who did not during the observation period. The mean dose was 22.5 mg/day in the OIC group and 17.8 mg/day in the non-OIC group. Although the OIC group tended to receive slightly higher opioid doses, the difference was not statistically significant.

The rationale for conducting this analysis was based on preliminary data aggregation, indicating that a substantial proportion of patients developed OIC even at relatively low opioid doses (≤10 mg/day); this dose typically corresponds to the initiation of opioid therapy in opioid-naïve patients [16,17]. We observed a numerical trend suggesting that the incidence of OIC increased with rising opioid doses. For instance, the incidence was 54.2% at doses <10 mg/day and increased to 80.8% at doses ranging from >40-80 mg/day. However, given that only a few patients (n=10) received opioid doses at the higher dose range, these findings should be interpreted with caution. Therefore, we investigated whether opioid dose correlates with the BFI score, an indicator of gastrointestinal motility. Although the regression line in the scatter plot showed an upward trend, the considerable variability in scores resulted in a low correlation coefficient (*r*=0.258), indicating that no clear correlation could be established.

We also used multivariate logistic regression to adjust for potential covariates that could confound the relationship between opioid dose and OIC incidence. The results indicated that for every 10 mg/day increase in opioid dose, the OR for the risk of developing OIC was 1.339. However, this association was not statistically significant, with a wide 95% CI (0.949‑1.890)* (p*=0.096), possibly due to the limited sample size and small number of events across subgroups, especially in the high-dose group receiving >40 mg/day opioids.

We only included patients who were not constipated before initiating opioid treatment. All patients with fewer than three bowel movements in the seven days before enrollment were excluded. Despite this, the development of OIC was observed in the majority of patients, including those receiving an opioid dose of 10 mg/day or less. In this study, no statistically significant correlation was observed between opioid dose and the incidence of OIC. Additionally, dose dependency was not clearly established, suggesting that opioids can induce OIC even at low doses (<10 mg/day). These findings underscore the need to manage OIC, regardless of opioid dose, and are consistent with previous studies [6,11,12].

Although not statistically significant, a trend was observed suggesting that higher opioid doses may be associated with an increased incidence of OIC. This may be attributed to the dose-dependent reduction in intestinal transport capacity caused by opioids [18]. The high incidence of OIC from the initial dose of opioids underscores the importance of prescribing laxatives concurrently with opioid initiation. This is consistent with the Japanese and European Union (EU) guidelines, which advocate the concurrent use of laxatives with opioids to effectively manage OIC [5,17]. Furthermore, other treatment options for OIC should be considered based on emerging evidence. A multicenter, double-blind, randomized, placebo‑controlled confirmatory trial demonstrated the preventive effect of naldemedine against OIC [19]. A recent retrospective study also reported that naldemedine was safe and effective in managing OIC in older patients with cancer aged ≥75 years [20].

Previous retrospective, single-institution cohort studies have explored the association between OIC and various potential risk factors. Poor general performance status, age ≥65 years, higher body mass index, and cancer-related cachexia have been identified as significant risk factors associated with a higher incidence of constipation [21,22]. However, a real-world, multicenter, observational cohort study reported that OIC was not associated with age, sex, cancer diagnosis, ECOG PS, or opioid dose [6]. In addition, a study of 94 patients receiving palliative care for constipation, most of whom had cancer, found no association between age and constipation [23]. In our study, we adjusted for opioid dose based on these potential factors to calculate the adjusted OR and minimize the possibility of confounding. We speculate that inter-individual variations in constipation among patients with cancer may be influenced by both genetic and non-genetic factors [11]. Although certain tumor types may impact bowel function, we did not include cancer type as a covariate in our analysis, as the previous study found no significant association [4].

Limitations

This study has several limitations. It did not assess the long-term effects of OIC, as data were collected only during the first 14 days following opioid initiation. Further research is needed to evaluate the long-term impact of OIC. Additionally, patients may have been aware of the risk of OIC through discussions with their physicians, which could have made them more attentive to bowel changes or prompted them to take preventive measures, such as increasing their fiber intake, exercising regularly, or improving hydration. Moreover, our results may be limited by potential confounding factors and some ambiguity in evaluation methods, leading to reporting variations. Though we adjusted for known relevant covariates in the multivariate logistic regression analysis, the effect of unmeasured and potential confounding factors, including variables such as diet, hydration, and genetic predisposition, which may have affected both OIC incidence and opioid dose, remains unknown. We did not use any model-building method for multivariate logistic regression analysis due to the small sample size in this study. Finally, limited sample size, especially in the subgroup of patients receiving >40 mg/day opioids, may have reduced the statistical power of this post hoc analysis.

## Conclusions

This post hoc analysis showed that OIC incidence is not significantly dependent on opioid dose. Notably, a substantial number of patients who did not have constipation at baseline developed OIC even at relatively low doses of opioids, implying that OIC can occur in any patient who initiates opioid therapy. Although there was a trend toward increased OIC incidence with higher opioid doses, it was not statistically significant. Future research should focus on assessing the long-term impact of OIC with large sample sizes. The high incidence of OIC even in patients with cancer receiving low-dose opioids highlights the need to manage OIC proactively, regardless of the opioid dose.
